# Dysregulation and functional roles of miR-183-96-182 cluster in cancer cell proliferation, invasion and metastasis

**DOI:** 10.18632/oncotarget.8715

**Published:** 2016-04-12

**Authors:** Yi Ma, A-Juan Liang, Yu-Ping Fan, Yi-Ran Huang, Xiao-Ming Zhao, Yun Sun, Xiang-Feng Chen

**Affiliations:** ^1^ Center for Reproductive Medicine, Ren Ji Hospital, School of Medicine, Shanghai Jiao Tong University, Shanghai, China; ^2^ Shanghai Key Laboratory for Assisted Reproduction and Reproductive Genetics, Shanghai, China; ^3^ Reproductive Medicine Center, Shanghai First Maternity and Infant Hospital, Tongji University School of Medicine, Shanghai, China; ^4^ Department of Urology, Ren Ji Hospital, School of Medicine, Shanghai Jiao Tong University, Shanghai, China

**Keywords:** miR-183-96-182 cluster, cancer development, cancer progression, metastasis, prognosis

## Abstract

Previous studies have reported aberrant expression of the miR-183-96-182 cluster in a variety of tumors, which indicates its' diagnostic or prognostic value. However, a key characteristic of the miR-183-96-182 cluster is its varied expression levels, and pleomorphic functional roles in different tumors or under different conditions. In most tumor types, the cluster is highly expressed and promotes tumorigenesis, cancer progression and metastasis; yet tumor suppressive effects have also been reported in some tumors. In the present study, we discuss the upstream regulators and the downstream target genes of miR-183-96-182 cluster, and highlight the dysregulation and functional roles of this cluster in various tumor cells. Newer insights summarized in this review will help readers understand the different facets of the miR-183-96-182 cluster in cancer development and progression.

## INTRODUCTION

microRNA (miRNA) is a small non-coding RNA molecule (containing about 22 nucleotides) that silences cognate target genes *via* base-pairing with complementary sequences within 3′UTRs (sometimes 5′UTRs or coding regions) in corresponding mRNAs, resulting in inhibition of translation or mRNA degradation [1]. miRNA is involved in various biological processes, including cell proliferation, apoptosis, metabolism and differentiation. miR-183-96-182 cluster is a highly conserved miRNA cluster [2]. Members of this cluster are located within a 5-kb region on human chromosome 7q32.2 [3], transcribed in the same direction from telomere to centromere, and have similar biological functions in some of the closely related signaling pathways.

The transcriptional start site (TSS) of miR-183-96-182 cluster has not yet been confirmed. Several studies suggested its localization in the 5207, 5200, or 5068 base upstream of miR-183 precursor [4–6]. Tang *et al*. have suggested that the potential TSS of miR-183-96-182 cluster maybe localized at the 5112 site upstream domain of miR-96, which contains seven binding domains of β-Catenin/TCF/LEF-1 complex [7]. Additionally, previous reports have indicated that three TGF-β response elements at 11519-9069 region upstream domain of miR-182, can directly interact with Smad2/Smad4 complex [8]. It is known, that many upstream regulators, including HSF2, β-catenin/TCF/LEF-1, TGF-β, SP1, P53, growth hormones, Akt/FOXP3, and MYOD increase the expression of miR-183-96-182 cluster [3, 7–18], while ZEB1, MYCN, HDAC, EVI1, and KLF-3 repress this cluster [9, 19–22]. Hypoxia and/or starvation are known to up-regulate miR-96/miR-182 expression, and miR-183/miR-182 increases the expression of hypoxia inducible factor 1α (HIF-1α) [23–27]. Thus, the relationship between miR-183-96-182 cluster and hypoxia or starvation still needs to be investigated.

While investigating its regulatory effect on the downstream target genes, the miR-183-96-182 cluster was discovered as being a regulator of tumor development, the nervous system and the immune system [2, 28–34]. Recent studies have documented localization of some tumor-related genes, such as CDK6, BRAF, and c-MET at the upstream/downstream domain of miR-183-96-182 cluster [23, 35, 36], suggesting that these genes might be regulated to process similar functions of tumor-related molecules. Furthermore, over-expression of miR-183-96-182 cluster has been described in most malignant tumors including, hepatocarcinoma [37–39], esophageal cancer [40], gastric carcinoma [7], prostate cancer [41], bladder cancer [42, 43], upper urinary tract urothelial cancer [44], colon cancer [45–47], lung cancer [48], breast cancer [3, 49], and chronic myeloid leukemia [50], indicating that it may function as an oncogene cluster. In contrast, miR-183-96-182 cluster functions as a tumor-suppressor gene with down-regulation in pancreatic cancer [51] and melanoma [52] have been documented. In addition, some recent studies have reported some contradicting features exhibited by the miR-183-96-182 cluster in gastric carcinoma [53, 54] and lung cancer [55–58].

The available evidence, thus, suggests much variability in the role played by the miR-183-96-182 cluster in tumorigenesis, tumor progression and metastasis. In this review, we profile the dysregulation and functional roles of the miR-183-96-182 cluster during tumorigenesis in various tumor cells, and its prognostic relevance in clinical settings. The outline of this paper is provided in Appendix 1-1.

## MATERIALS AND METHODS

In this review, we performed an online search of articles published from January 2000 to March 2016 in Pubmed (http://www.ncbi.nlm.nih.gov/pubmed). We used the following query: (miR-96 OR miR96 OR microRNA96 OR miR-183 OR miR183 OR microRNA183 OR miR-182 OR miR182 OR microRNA182 OR miR-183-96-182 OR miR-183/96/182 OR miR-183~96~182). Only English language articles were included. A total of 620 records were retrieved. We then reviewed the titles and abstracts, and eliminated duplicate and irrelevant articles. Eventually, 155 full-length articles were included in this review.

## RESULTS AND DISCUSSION

### miR-183-96-182 cluster in cancer cell proliferation

#### miR-183-96-182 cluster promotes cancer cell proliferation

For examining the role of miR-183-96-182 cluster in cell proliferation, the relation between miR-96 and the members of forkhead box protein (FOX) family has been investigated. FOXO, a subfamily of FOX family that includes FOXO1, FOXO2, FOXO3 and FOXO4, was found to be associated with cell apoptosis. Recent studies have demonstrated that FOXO can activate Bim and p27kip1, resulting in increased cell apoptosis and cell cycle inhibition [59, 60]. In 2009, Guttilla *et al.* found coordinated repression of FOXO1 by miR-96, miR-182 and miR-27a in breast cancer cells [49]. Targetscan prediction revealed three members of the FOXO protein family, including FOXO1, FOXO3 and FOXO4 as potential targets of the miR-183-96-182 cluster. However, only FOXO1 and FOXO3 have been confirmed by previous studies [16, 35, 49, 61–65], in various types of cancers such as, prostate [63, 66, 67], bladder [43, 68], colorectal [62], breast [69], lung [61], lymphoma [64], and endometrial carcinoma [70] (Table 1). Additionally, recent studies have indicated that miR-182 promotes cell proliferation and tumor invasion by targeting FOXF2 [71, 72], a known inhibitor of MMPs and WNT5A [73, 74].

**Table 1:** miR-183-96-182 cluster in cancer cell proliferation

Member of miR-183-96-182 cluster	Oncogene/Tumor suppressor	Target genes	Tested in human cancer tissue	Cell lines	Cancer types	Results
miR-183-96-182	Oncogene	FOXO1	--	L428	Lymphoma	Promotes cell proliferation [64].
miR-183-96-182	Oncogene	FGF9, CPEB1, FOXO1	✓	U251, U87	Glioma	Promotes cell growth [65].
miR-183-96-182	Oncogene	RAB21(miR-183) RAB40B(miR-96 and miR-183) TNFSF11(miR-96)		MCF-7, T47D	Breast cancer	Promotes cell proliferation [3].
miR-183-96-182	Oncogene	--	✓	T24, UM-UC-3	Bladder Cancer	Promotes cell proliferation [154].
miR-183-96-182	Oncogene	--	--	R262, R300 UW402, UW426, D341, D384, D425, D458, D556, D283, DAOY	Medulloblastoma	Promote cell proliferation [155].
miR-96, miR-182	Oncogene	FOXO1	✓	MCF-7, T47D, MDA-MB-231, MDA-MB-435	Breast cancer	Promote cell proliferation [49].
miR-96, miR-182	Oncogene	EFNA5	✓	HepG2, Hep3B, Huh7, SK-Hep1	Hepatocellular carcinoma	Promote cell proliferation [85].
miR-96, miR-182	Tumor suppressor	-	--	A375, SK-MEL-28	Melanoma	Inhibit cell proliferation [52]
miR-183	Oncogene	PDCD4	✓	Eca109, TE13 and EC109, EC9706	Esophageal cancer	Promote cell proliferation [76, 81].
miR-183	Oncogene	PDCD4	✓	SGC-7901	Gastric cancer*	Promote cell proliferation [78].
miR-183	Oncogene	NEFL	✓	U251	Glioma	Promote cell proliferation [156].
miR-183	Oncogene	PDCD4	✓	HepG2, Huh7	Hepatocellular carcinoma	Promote cell proliferation [79].
miR-183	Oncogene	PDCD4	--	SW1990	Pancreatic cancer	Promote cell proliferation [80].
miR-183	Oncogene	SOCS-6	✓	PANC-1	Pancreatic cancer	Promote cell proliferation [157].
miR-183	Oncogene	SOCS-6	✓	HepG2, Hep3B	Hepatocellular carcinoma	Promote cell proliferation [158].
miR-183	Oncogene	PP2A-Cα, PP2A-Cβ, PP2A-B56-γ	✓	ACHN, A498	Renal cancer	Promote cell proliferation [104].
miR-183	Oncogene	DKK-3, SMAD4	✓	PC-3, DU-145, LNCaP	Prostate cancer	Promote cell proliferation [159].
miR-183	Tumor suppressor	BMI1	✓	AGS, SGC7901, MKN28, MGC803, HGC27	Gastric cancer*	Inhibit cell proliferation [103].
miR-96	Oncogene	FOXO1	✓	HEC-1B	Endometrial cancer.	Promote cell proliferation [70].
miR-96	Oncogene	FOXO1	--	HepG2	Hepatocellular carcinoma	Promote cell proliferation [160].
miR-96	Oncogene	FOXO1	✓	T24	Bladder cancer	Promote cell proliferation [68].
miR-96	Oncogene	FOXO1	✓	PC3, LNCaP, and LNCaP, DU-145, PC3, 22rv-1, and 22Rv1, LNCaP clone FGC, DU145, PC3	Prostate cancer**	Promote cell proliferation [63, 66, 67].
miR-96	Oncogene	FOXO1	✓	SW480, SW620	Colorectal cancer	Promote cell proliferation [62].
miR-96	Oncogene	FOXO3	--	HepG2	Hepatocellular carcinoma	Promote cell proliferation [160].
miR-96	Oncogene	FOXO3	✓	SW480, SW620	Colorectal cancer	Promote cell proliferation [62].
miR-96	Oncogene	FOXO3	✓	MCF-7, ZR-75-30, BT549, Bcap37, MDA-MB435, SKBR3, MDA-MB453, T47D	Breast cancer	Promote cell proliferation [69].
miR-96	Oncogene	FOXO3	✓	A549, SPC-A-1	Lung cancer***	Promote cell proliferation [61].
miR-96	Oncogene	RECK	✓	MDA-MB-231, MCF-7, MDA-MB-468, MDA-MB-435, T-74D, MDA-MB-453	Breast cancer	Promote cell proliferation [83].
miR-96	Oncogene	RECK	✓	A549, SK-MES-1, H1299	Lung cancer***	Promote cell proliferation [84].
miR-96	Oncogene	HBP1	✓	U-87 MG, U-251 MG, U-373 MG, M059J	Glioma	Promote cell proliferation [75].
miR-96	Oncogene	MTOR	✓	LNCaP, 22Rv-1	Prostate cancer	Promote cell proliferation (Under hypoxia) [23].
miR-96	Tumor suppressor	KRAS	✓	HPDE, BxPC-3, PK-8, and MIA PaCa-2, PANC-1, BxPC-3	Pancreatic cancer	Inhibit cell proliferation [20, 51].
miR-96	Tumor suppressor	HERG1	✓	PANC-1, SW1990, CFPAC-1, HPAC, BxPC-3	Pancreatic cancer	Inhibit cell proliferation [89].
miR-96	Tumor suppressor	GPC1	✓	Panc-1, AsPC-1, BxPC-3	Pancreatic cancer	Inhibit cell proliferation [90].
miR-96	Tumor suppressor	ALK	✓	Karpas 299, SUP-M2, SU-DHL-1, SR-786, DEL, SH-SY5Y, H2228	Lymphoma, Neuroblastoma, and lung *** cancer	Inhibit cell proliferation [88].
miR-96	Tumor suppressor	ATG7	✓	LNCaP, 22rv-1	Prostate cancer**	Inhibit cell proliferation (Under hypoxia) [23].
miR-96	Tumor suppressor	REV1, RAD51	--	U2OS, HeLa, HCC1937, MDA-MB-231, HCT116, PEO1, PEO1 C4-2	Multiple tumors	Sensitize cancer cells to cisplatin and PARP inhibition [36].
miR-182	Oncogene	PDCD4	--	A549, SPC-A-1 and A549	Lung cancer****	Promote cell proliferation [55, 56].
miR-182	Oncogene	PDCD4	✓	OVCAR3, SKOV3, OV2008, HEY, 3AO, A2780, HO8910, C13	Ovarian cancer	Promote cell proliferation [77].
miR-182	Oncogene	CHL1	✓	TPC-1, BCPAP	Papillary thyroid carcinoma	Promote cell proliferation [161].
miR-182	Oncogene	SATB2	✓	DLD-1, HCT116, SW480, SW620, Lovo	Colorectal cancer	Promote cell proliferation [162].
miR-182	Oncogene	FOXF2	✓	HT29, SW480, SW620, HCT116	Colorectal cancer	Promote cell proliferation [71].
miR-182	Oncogene	-	✓	HCT116, HT29, SW480	Colon cancer	Promote cell proliferation [163].
miR-182	Oncogene	CEBPA	✓	--	Hepatocellular carcinoma	Promote cell proliferation [164].
miR-182	Oncogene	TP53INP1	✓	HEK293, HepG2	Hepatocellular carcinoma	Promote cell proliferation [86].
miR-182	Oncogene	LRRC4	✓	U251, SF126, SF767	Glioma	Promote cell proliferation [165].
miR-182	Oncogene	TCEAL7	--	HEC-1B, RL95-2, AN3CA	Endometrial carcinoma	Promote cell proliferation [166].
miR-182	Oncogene	CUL5	✓	Ishikawa H	Endometrial carcinoma	Promote cell proliferation [167].
miR-182	Oncogene	NDRG1	✓	LNCap, PC-3, DU145, 22Rv1	Prostate cancer******	Promote cell proliferation [126].
miR-182	Oncogene	FOXF2, RECK, MTSS1	✓	LNCaP, PC-3, DU145	Prostate cancer******	Promote cell proliferation [72].
miR-182	Oncogene	PFN1	✓	MDA-MB-231	Breast cancer	Promote cell proliferation [168].
miR-182	Oncogene	RECK, Smad4	✓	J82, T24, UM-UC-3	Bladder cancer	Promote cell proliferation [169].
miR-182	Oncogene	FOXO3	--	A549, H1299, CL 1-0, CL 1-5	Lung cancer****	Promote cell proliferation [16].
miR-182	Tumor suppressor	RAD51	--	OCI-AML3, MV4-11	Acute myelogenous leukemia.	Sensitize cancer cells to sapacitabine [21].
miR-182	Tumor suppressor	MITF, BCL2, cyclin D2	--	M23, SP6.5	Posterior uveal melanoma	Inhibit cell proliferation [102].
miR-182	Tumor suppressor	RGS17	--	CRL-5803, CRL-5889, H2126	Lung cancer****	Inhibit cell proliferation [57].
miR-182	Tumor suppressor	RASA1	✓(highly expressed in tumor)	Failed to get to the data	Lung squamous cell carcinoma	Inhibit cell proliferation [58].
miR-182	Tumor suppressor	CTTN	--	A549	Lung cancer****	Inhibit cell proliferation [153].
miR-182	Tumor suppressor	ANUBL1	✓	SGC-7901	Gastric cancer	Inhibit cell proliferation [170].
miR-182	Tumor suppressor	CREB1	✓	MGC-803, BGC-823, SGC-7901	Gastric cancer	Inhibit cell proliferation [53].
miR-182	Tumor suppressor	FLOT1	✓	786-O, Caki-1	Renal cell carcinoma	Inhibit cell proliferation [101].
miR-182	Tumor suppressor	BCL2, P21(Not biologically validated)	--	PC3, LNCaP	Prostate cancer******	Inhibit cell proliferation [25].

* Contradictory findings were marked as the same number of *.

Abbreviations: ALK: Anaplastic lymphoma kinase; ANUBL1: Zinc finger, AN1-type domain 4; ATG7: Autophagy related 7; BCL2: B-cell CLL/lymphoma 2; BMI1: BMI1 proto-oncogene; BRCA1: Breast cancer 1 (BRCA1); CEBPA: CCAAT/enhancer binding protein (C/EBP), alpha; CHL1: Cell adhesion molecule L1-like; CREB1: cAMP-responsive element binding protein 1; CTTN: Cortactin; CUL5: Cullin-5; DAP12: DNAX activating protein 12 kDa; DKK-3: Dickkopf homolog-3; EFNA5: EphrinA5, FOXO: Forkhead box O; FOXF2: Forkhead box F2; FGF9: Fibroblast growth factor 9; GPC1: Glypican 1; HBP1: HMG-box transcription factor 1; HERG1: Human ether-a-go-go-related potassium channel; IDH2: Isocitrate dehydrogenase 2; KRAS: Kirsten rat sarcoma viral oncogene homolog; LRRC4: Leucine rich repeat containing 4; MITF: Microphthalmia-associated transcription factor; MTOR: Mechanistic target of rapamycin; MTSS1: Metastasis suppressor 1; NDRG1: N-myc downstream regulated 1; NDRG1: N-myc downstream regulated gene 1; P21: Cyclin-dependent kinase inhibitor 1A; PDCD4: Programmed cell death 4; PFN1: Profilin 1; PP2A: Protein phosphatase 2A; RASA1: RAS p21 GTPase activating protein 1; RECK: Reversion-inducing-cysteine-rich protein with kazal motifs; RAB: Ras-related gtp-binding protein, alternative splice; RAD51: RAD51 recombinase; REV1: REV1, polymerase; RGS17: Regulator of G-protein signaling 17; SATB2: SATB homeobox 2; SMAD4: SMAD family member 4; TCEAL7: Transcription elongation factor A-like 7; TNFSF11: Tumor necrosis factor (ligand) superfamily, member 11; TP53INP1: Tumor protein p53 inducible nuclear protein 1.

HMG-box transcription factor 1 (HBP-1), the target gene of miR-96, has been shown to inhibit Wnt/β-Catenin signaling pathway, and suppress cell proliferation and survival. Thus, miR-96 appears to promote tumor cell growth by down-regulation of HBP-1 in glioma cells [75]. Furthermore, the activation of β-Catenin/TCF/LEF-1 signaling pathway, which is stimulated by knock-down of glycogen synthase kinase 3 beta (GSK3β) [7], is known to induce up-regulation of miR-96 expression [7, 13]. As a serine/threonine protein kinase, GSK3β is essential for NF-κB-mediated anti-apoptotic response. Knock-down of GSK3β expression induces up-regulation of β-Catenin/TCF/LEF-1 complex, which binds to the promoter of miR-183-96-182 cluster and stimulates its transcription. Thus, up-regulation of the miR-183-96-182 cluster *via* GSK3β-mediated β-Catenin/TCF/LEF-1 signaling pathway can promote abnormal cell proliferation in gastric cancer [7]. The schematic diagram is provided in Appendix 1-2.

Previous studies have revealed that miR-183 and miR-182 promote cell proliferation, tumor invasion, and chemo-resistance by inhibition of programmed cell death 4 (PDCD4) in various cancer cells [55, 56, 76–81]. As a typical tumor suppressor gene (TSG), PDCD4 can inhibit eukaryotic translation initiation factor 4A1 (EIF4A1) and NF-κB-dependent transcriptional factors *via* direct interaction with p65, to induce apoptosis in glioblastoma cells [82]. The PDCD4-targeted inhibition by the miR-183-96-182 cluster, described in various cancers, is summarized in Table 1. Notably, miR-96 has also been found to inhibit the TSG RECK [40, 83, 84] and EFNA5 [85]. Besides, miR-96 and miR-182 were found to have an inhibitory effect on TP53INP1 expression [62, 86]. Collectively, the available evidence indicates that miR-183-96-182 cluster could promote cell proliferation in various cancer types (Table 1).

#### miR-183-96-182 cluster inhibits cancer cell proliferation

Interestingly, in certain cancers, over-expression of miR-183-96-182 cluster had an inhibitory effect on cell proliferation, a finding which is not consistent with the earlier reports related to most cancer types. The miR-96 target gene, ATG7, is a key factor in the autophagy pathway, which protects the cancer cells against stress responses such as hypoxia or starvation [87]. High-expression of miR-96 is thought to inhibit autophagy through directly targeting ATG7, and subsequently inhibit the survival of cancer cells under hypoxic conditions [23]. In addition, miR-96 is known to down-regulate RAD51 (a DNA repair protein) and REV1 (a DNA polymerase) to promote cellular sensitivity to cisplatin, which binds to and cause crosslinking of DNA to ultimately trigger apoptosis [36]. Similar results were also found for miR-182 in acute myelogenous leukemia [21]. Thus, the over-expression of miR-96/miR-182 appears to dramatically promote drug sensitization in cancer cells [36]. miR-96 was also shown to inhibit cell proliferation of ALK-expressing cancer cells *via* suppressing ALK expression, as well as those ALK-targeted genes, including AKT, STAT3, JNK and IGF-1 [88].

Notably, the inhibitory effect of miR-96 on pancreatic cancer cell proliferation has been clearly elucidated in the past few years [20, 51, 89, 90]. In pancreatic cancer, three important oncogenes, including KRAS [51], human either a go-go-related gene type 1 (HERG1) [89] and Glypican 1 (GPC1) [90], are known to be miR-96 target genes. KRAS, aberrantly activated in approximately 90% of pancreatic cancers [91], can promote abnormal cell proliferation by activating PI3K/Akt, NF-κB and ERK signaling pathways [92–95]. HERG1 is over-expressed in various cancer cells and found to promote cell proliferation [96–98]. GPC1 is exhibited high-expression in pancreatic cancers for efficient proliferation and angiogenesis [99]. miR-96 is known to target these three genes and, thereby, significantly increase the apoptosis rate in pancreatic cancer cells. However, the biological functions of miR-183 and miR-182 in pancreatic cancer are still unclear [80, 100]. Similar inhibitory effects were also observed in renal cell cancinoma and melanoma [52, 101, 102]. In contrast to the usual oncogenic function of the miR-183-96-182 cluster in most cancer types, the above tumor suppressor activity suggests a specific context (hypoxia/chemotherapy), phenotype, or cancer cell-dependent regulation of the miR-183-96-182 cluster in tumorigenesis.

#### Contradictory results

However the functions of miR-183-96-182 cluster in lung and gastric cancer are yet to be confirmed (Table 1). In non-small cell lung cancer, miR-96 was shown to promote cell proliferation by targeting FOXO3 and RECK mRNA (A549, SK-MES-1, H1299 and SPC-A-1 cell lines) [61, 84], while according to a study by Vishwamitra *et al.*, miR-96 inhibits cell proliferation by targeting ALK (H2228 cell line) [88]. This reported discrepancy in results may be attributable to the inclusion of different cell types for analysis or involvement of different signaling pathways. Two studies on gastric carcinoma simultaneously reported contradictory results with respect to the function of miR-183 during cell proliferation in SGC-7901 cells [78, 103], Xu *et al*. found miR-183 was down-regulated in 65 gastric cancer tissue and 5 gastric cancer cell lines, miR-183 significantly inhibited SGC7901 and AGS cell viability with MTT assay. In contrast, Gu *et al*. found miR-183 was up-regulated in 80 tumor tissue, miR-183 significantly promoted SGC7901 cell proliferation by MTT and flow cytometry assay. These findings suggest that the regulation of cell proliferation by miR-183-96-182 cluster is a complicated synergic process, and the different functions of this cluster may be due to that the target genes might be expressed at different levels, contain mutations, or compete with other molecules. Other possible reasons for the contradictory results are summarized in Appendix 1-3.

### miR-183-96-182 cluster in tumor invasion and metastasis

#### miR-183-96-182 cluster promotes tumor invasion and metastasis

It has been demonstrated that miR-183-96-182 cluster promotes tumor invasion and metastasis in most cancers, including thyroid, esophagus, gallbladder, ovary, bladder, kidney, liver cancers, melanoma, medulloblastoma, sarcoma, glioma, and myeloid cell tumor (Table 2). miR-183 promotes tumor invasion and metastasis by targeting PDCD4, protein phosphatase 2A (PP2A), EGR1 and PTEN [76, 78, 104, 105]. In addition, TGF-β and Smad can also promote prostate cancer bone metastasis by induction of miR-96 and activation of the mTOR pathway [106]. Moreover, the inhibitory effect on metastasis in hepatoma carcinoma cells, induced by the suppression of miR-96 [107], was reported as being associated with the inhibition of EFNA5 expression by miR-96-targeting [85]. Similar findings have been reported in case of gastric, bladder, and breast cancers [3, 7, 83, 108]. With regard to miR-182, Huynh *et al.* reported significant suppression of invasive growth tendency and metastasis by suppressing miR-182 *in vivo* [109]. Moreover, similar to the effects of TGF-β on miR-96, TGF-β up-regulates miR-182, which can target CYLD and thus promote the activation of NF-κB in gliomablastoma. Therefore, TGF-β-mediated up-regulation of miR-182 probably results in the persistent activation of NF-κB in gliomblastoma, which subsequently leads to angiogenesis and tumor invasion. Table 2 shows the target genes of miR-183-96-182 cluster which regulate invasion and metastasis in various tumor cells.

**Table 2:** miR-183-96-182 cluster in tumor invasion, migration, and metastasis

Member of miR-183-96-182 cluster	Oncogene/Tumor suppressor	Target genes	Cell lines	Cancer types	Results
miR-183-96-182	Oncogene	RAB21(miR-183) RAB40B(miR-96 and miR-183) TNFSF11(miR-96)	MCF-7, T47D	Breast cancer*	Promote migration [3].
miR-183-96-182	Oncogene	BRMS1L	MCF-7, T47D, MDA-MB-435s, MDA-MB-468	Breast cancer*	Promote EMT and invasion [15].
miR-183-96-182	Oncogene	--	R262, R300 UW402, UW426, D341, D384, D425, D458, D556, D283, DAOY	Medulloblastoma	Promote migration [155].
miR-183-96-182	Oncogene	FOXO1	Hep3B, SNU387, HKCI-1, HKCI-8	Hepatocellular carcinoma	Promote migration [13].
miR-183-96-182	Tumor suppressor	FOXF2	55 human NSCLC cell lines	Lung cancer	Inhibit invasion and metastasis [116].
miR-183, miR −96	Tumor suppressor	SLUG, ZEB1, ITGB1, and KLF4	HCT116, MCF10A	Colon cancer***	Inhibit EMT, migration, and invasion [9].
miR-96, miR-182	Oncogene	EFNA5	HepG2, Hep3B, Huh7, SK-Hep1	Hepatocellular carcinoma	Promote invasion [85].
miR-183	Oncogene	--	HTori-3, FTC-133	Follicular thyroid carcinomas	Promote migration [171].
miR-183	Oncogene	PDCD4	Eca109, TE13	Esophageal cancer	Promote invasion [76].
miR-183	Oncogene	PDCD4	SGC-7901	Gastric cancer**	Promote invasion [78].
miR-183	Oncogene	PDCD4	SW1990	Pancreatic cancer	Promote invasion and migration [80].
miR-183	Oncogene	SOCS-6	PANC-1	Pancreatic cancer	Promote invasion and metastasis [157].
miR-183	Oncogene	SOCS-6	HepG2, Hep3B	Hepatocellular carcinoma	Promote invasion [158].
miR-183	Oncogene	PP2A-Cα, PP2A-Cβ, and PP2A-B56-γ	ACHN, A498	Renal cancer	Promote migration and invasion [104].
miR-183	Oncogene	EGR1 and PTEN	SYO-1, FUJI, HCT116, DLD1, Rh30, JR1	Synovial sarcoma, RMS, and colon*** cancer	Promote migration [105].
miR-183	Oncogene	NEFL	U251	Glioma	Promote invasion [156].
miR-183	Tumor suppressor	TIAM1	SKOV-3ip, HO-8910PM	Ovarian cancer	Inhibit migration and invasion [124].
miR-183	Tumor suppressor	BMI1	AGS, SGC7901, MKN28, MGC803, HGC27	Gastric cancer**	Inhibit invasion [103].
miR-183	Tumor suppressor	EZR	MGC-803, SGC-7901, BGC-823, MKN-45, MKN-28	Gastric cancer**	Inhibit invasion [120].
miR-183	Tumor suppressor	EZR	SOSP-9607, and MG63, U2OS, Saos2, HOS, SV40	Osteosarcoma	Inhibit migration and invasion [118, 119].
miR-183	Tumor suppressor	EZR	MDA-MB-231, T47D, SKBR-3, ZR-75-1	Breast cancer*	Inhibit migration [121].
miR-183	Tumor suppressor	EZR	801D, 95C	Lung cancer	Inhibit migration [122].
miR-183	Tumor suppressor	MMP-9	Siha, HeLa	Cervical carcinoma	Inhibit invasion and metastasis [172].
miR-183	Tumor suppressor	ITGB1 and KIF2A	HeLa	Cervical carcinoma	Inhibit migration and invasion [125].
miR-96	Oncogene	RECK	MDA-MB-231, MCF-7, MDA-MB-468, MDA-MB-435, T-74D, MDA-MB-453	Breast cancer	Promote invasion [83].
miR-96	Oncogene	MAP4K1 and IRS1	T24	Bladder cancer	Promote invasion [108].
miR-96	Oncogene	--	AGS	Gastric cancer	Promote invasion [7].
miR-96	Oncogene	--	HCCLM6	Hepatocellular carcinoma	Promote invasion [107].
miR-96	Oncogene	AKT1S1	DU145, PC3, LNCap, 22Rv1, RasB1, AC1, AC3	Prostate cancer	Promote bone metastasis [106].
miR-96	Tumor suppressor	KRAS	HPDE, BxPC-3, PK-8, and MIA PaCa-2, PANC-1, BxPC-3	Pancreatic cancer	Inhibit migration and invasion [20, 51].
miR-182	Oncogene	MTSS1	HLE, HLF, HepG2, Hep3B, HUH-1	Hepatocellular carcinoma	Promote invasion [173].
miR-182	Oncogene	CYLD	LN382T, A172, T98G, LN18, LN229, LN464, SNB19, U373MG, U87MG, LN444, LN443, LN428, U118MG, LN-Z308, LN319	Glioma	Promote invasion [8].
miR-182	Oncogene	RECK	MCF-7, MDA-MB-231, SKBR3, BT-20	Breast cancer	Promote tumorigenicity and invasion [12].
miR-182	Oncogene	MIM	4T1 series, MCF10 series	Breast cancer	Promote invasion and metastasis [174].
miR-182	Oncogene	PFN1	MDA-MB-231	Breast cancer	Promote invasion [168].
miR-182	Oncogene	--	DAOY, D458 Med, Med8A	Medulloblastoma	Promote migration [175].
miR-182	Oncogene	RSU1, MTSS1, PAI1, and TIMP1	STS-48, STS-109, STS-145, primary mice sarcomas cell lines (Kras and p53 mutation)	Sarcomas	Promote migration, invasion and metastasis [176].
miR-182	Oncogene	--	Primary mice sarcomas cell lines	Sarcomas	Promote metastasis [18].
miR-182	Oncogene	CHL1	TPC-1, BCPAP	Papillary thyroid carcinoma	Promote invasion [161].
miR-182	Oncogene	SATB2	DLD-1, HCT116, SW480, SW620, Lovo	Colorectal cancer	Promote migration, invasion and metastasis [162].
miR-182	Oncogene	FOXF2	HT29, SW480, SW620, HCT116	Colorectal cancer	Promote invasion [71].
miR-182	Oncogene	TSP-1	HCT-116, HT-29	Colon cancer	Promote metastasis [177].
miR-182	Oncogene	PDCD4	A549	Lung cancer****	Promote invasion [56].
miR-182	Oncogene	PDCD4	OVCAR3, SKOV3, OV2008, HEY, 3AO, A2780, HO8910, C13	Ovarian cancer	Promote invasion [77].
miR-182	Oncogene	BRCA1, MTSS1, and HMGA2	SKOV3, HEY, OVCAR-3	Ovarian cancer	Promote invasion and metastasis [178].
miR-182	Oncogene	MITF and FOXO3	SK-MEL-19, -29, -85, -94, -100, -103, -147, -173, -187, -192, -197. 501mel, B16F10, WM35	Melanoma	Promote migration, invasion and metastasis [35].
miR-182	Oncogene	--	--	Melanoma	Promote metastasis [109]
miR-182	Oncogene	CADM1	GBC-SD	Gallbladder cancer	Promote migration, invasion and metastasis [11].
miR-182	Oncogene	NDRG1	LNCap, PC-3, DU145, 22Rv1	Prostate cancer*****	Promote invasion [126].
miR-182	Oncogene	FOXF2, RECK and MTSS1	LNCaP, PC-3, DU145	Prostate cancer*****	Promote invasion [72].
miR-182	Oncogene	RECK and SMAD4	J82, T24, UM-UC-3	Bladder cancer	Promote invasion and metastasis [169].
miR-182	Tumor suppressor	FOXO3	A549, H1299, CL 1-0, CL 1-5	Lung cancer****	Inhibit migration and invasion [16].
miR-182	Tumor suppressor	GNA13	PC3, LNCaP	Prostate cancer*****	Inhibit invasion [123].

* Contradictory findings were marked as the same number of *.

Abbreviations: AKT1S1: AKT1 substrate 1; BMI1: BMI1 proto-oncogene; BRCA1: Breast cancer 1, early onset; BRMS1L: Breast Cancer Metastasis Suppressor 1-like; CADM1: Cell adhesion molecule 1; CHL1: Cell adhesion molecule L1-like; CYLD: Cylindromatosis; EGR1: Early growth response 1; EZR: Ezrin; FOXO: Forkhead box O; FOXF2: Forkhead box F2; GNA13: G-protein subunit α-13; HMGA2: High mobility group AT-hook 2; IRS1: Insulin receptor substrate 1; ITGB1: Integrin, beta 1; KIF2A: Kinesin heavy chain member 2A; KLF4: Kruppel-like factor 4; KRAS: Kirsten rat sarcoma viral oncogene homolog; MAP4K1: Mitogen-activated protein kinase kinase kinase kinase 1; MIM: Missing in Metastasis; MMP-9: Matrix metallopeptidase 9; MITF: Microphthalmia-associated transcription factor-M; MTSS1: Metastasis suppressor 1; NDRG1: N-myc downstream regulated 1; PDCD4: Programmed cell death 4; PFN1: profilin 1; PP2A: Protein phosphatase 2A; PTEN:Phosphatase and tensin homolog; RECK: Reversion-inducing-cysteine-rich protein with kazal motifs; PAI1:; Plasminogen activator inhibitor-1; RSU1: Ras suppressor protein 1; SATB2: SATB homeobox 2; SLUG: Snail family zinc finger 2; SMAD4: SMAD family member 4; SOCS-6: Suppressor of cytokine signaling 6; TIAM1: T-cell lymphoma invasion and metastasis 1; TIMP1: Tissue inhibitor of metalloproteinases 1; TSP-1: Thrombospondin-1; ZEB1: Zinc finger E-box binding homeobox 1

#### miR-183-96-182 cluster inhibits tumor invasion and metastasis

On the contrary, miR-183-96-182 suppresses tumor metastasis in lung, colon, and pancreatic cancers (Table 2). Transcriptional repressor Zin C finger E-box-binding homeobox 1 (ZEB1) family is a series of transcription factors which contain zinc finger domain. The highly conserved zinc finger structure can bind to E-box domain of the promoter of target genes, such as E-cadherin, the key epithelial marker for epithelial-mesenchymal transition (EMT and MET) [110, 111]. A recent study indicated a ZEB1/miR-200 double negative feedback loop in EMT at different stages of tumor development [112]. Notably, miR-183/96 can inhibit EMT *via* suppressing ZEB1 expression. Besides, ZEB1 can also block the transcription of miR-183-96-182 cluster by binding to its promoter [9]. miR-183-96-182 cluster and ZEB1 exert a double negative feedback loop in p21−/− cells. However, more recently, p21, an inhibitor of cyclin-dependent kinase through suppressing the expressions of CDK1 and CDK1 proteins [113], can also inhibit EMT progression [114, 115]. There is further evidence that p21 can interact with ZEB1 to form a complex and binds to the promoter of miR-183-96-182 cluster, which suppresses the transcription inhibition by ZEB1 and results in the suppression of EMT. The schematic diagram is provided in Appendix 1-4. Similar results were also reported in lung cancer cells by Kundu *et al.*, where they found that FOXF2 correlates with ZEB1 expression, and miR-183-96-182 can suppress FOXF2 to inhibit tumor invasion and metastasis in lung cancers [116].

The EZR gene, the target gene of miR-183, plays an important role in angiogenesis and tumor metastasis in various tumors [117]. The miR-183 was found to block MAPK/ERK signaling pathway, as well as inhibit tumor invasion and metastasis by suppressing EZR expression in gastric, breast, lung cancers, and osteosarcoma [118–122]. Additionally, several previous studies demonstrated that some oncogenes, including TIAM1, BMI1, TSP-1, FOXO3, GNA13, ITGB1, KIF2A, SLUG, ITGB1, and KLF4, were targeted by miR-183 and miR-96 for the suppression of invasion and metastasis in oophoroma, lung, prostate, colon, cervix, stomach and pancreas cancer cells [9, 16, 20, 51, 103, 123–125] (Table 2).

#### Contradictory results

Investigations of the effects of miR-183-96-182 cluster on tumor invasion and metastasis have sometimes yielded contradictory results in different tumors, and in some cases, even within the same tumor type. miR-183 was found to be down-regulated by Cao *et al*. (in 52 pairs of FFPE samples and 5 cell lines) and Xu *et al*. (in 65 pairs of samples and 5 cell lines) (Table 2) and hypothesized to inhibit tumor invasion by suppressing the expressions of BMI1 or EZR proteins in gastric cancers [103, 120]. Conversely, Hu *et al.* reported that miR-183 was up-regulated (20 non-tumor tissue and 80 tumor tissue samples) and promotes gastric cancer cell invasion by inhibiting PDCD4 expression [78]. Similar differences in results were also reported in case of prostate cancers. miR-182 was over-expressed in prostate cancer tissue by Hirata *et al*. (52 paired samples) and Liu *et al*. (5 tumor and 3 non-tumor tissue) and enhanced the invasive and migratory capacity in PC3 and DU145 cells by targeting NDRG1, FOXF2, RECK, and MTSS1 genes [72, 126]. In contrast, over-expression of miR-182 was shown to inhibit tumor invasion in PC3 and LNCaP cells by suppressing GNA13 expression [123]. These findings suggest a context-dependent phenotype for the miR-183-96-182 cluster in carcinogenesis which needs to be further investigated to understand the complex interactions, especially in those cancers where contradictory results have been observed, such as prostate, colon, lung, breast, and gastric cancers (Table 2).

### miR-183-96-182 cluster in cancer prognosis

Most of cancer cells display high-expression of miR-183 [127, 128]. The up-regulation of miR-183 is known to be associated with poor prognosis in breast cancer, colorectal cancer, hepatocellular cancer, and prostate cancer [13, 129–134], while predicts a good prognosis in osteosarcoma [135] (Table 3). This finding is consistent with its functions in cell proliferation, invasion and metastasis in these tumors types. Notably, miR-183 might affect the prediction for PSA-dependent diagnosis and prognosis *via* regulating PSA expression [136]. With respect to the prediction of miR-183-related prognosis, the available evidence from different studies is contradictory in lung cancer. Lin *et al.* showed the low expression of miR-183 in the peripheral blood which was associated with increased TNM stage in lung cancer patients (13 squamous-cell carcinoma and 17 adenocarcinoma) [137]. While Zhu *et al.* demonstrated the up-regulation of miR-183 family in lung cancer tissue (36 squamous-cell carcinoma and 34 adenocarcinoma), and that it appeared to confer a poor prognosis [48]. The wide variability in the reported results may be attributable to the differences between blood and tissue or the heterogeneity in lung cancer cells.

**Table 3:** miR-183-96-182 cluster for prediction of cancer prognosis

Member of miR-183-96-182 cluster	Oncogene/Tumor suppressor	Cancer types	Target genes	Results
miR-183-96-182	Oncogene	Hepatocellular carcinoma (tissue)	FOXO1	Associated with prognosis (microvascular invasion, tumor differentiation, and patients survival) [13].
miR-183-96-182	Oncogene	Lung cancer (tissue and serum)	--	Associated with prognosis (survival) [48].
miR-183, miR-96	Oncogene	Prostate cancer (tissue)	--	Associated with prognosis (tumor aggressiveness, metastatic and overall survival) when combined with other microRNAs [131].
miR-183	Oncogene	Lung cancer (tissue)*	--	Associated with prognosis (lymph node metastasis, clinical stage and EGFR mutation and patients survival) [179].
miR-183	Oncogene	Breast cancer (tissue)	--	Associated with prognosis (TNM clinical stage) [129].
miR-183	Oncogene	Colorectal cancer (plasma)	--	Associated with cancer recurrence and prognosis (lymph node metastasis, distant metastasis, TNM stage) [132].
miR-183	Oncogene	Colorectal cancer (tissue)	--	Associated with prognosis (clinical stage, lymph node metastasis, distant metastasis and patients survival) [133].
miR-183	Oncogene	Hepatocellular carcinoma (tissue)	--	Associated with cancer progression (TNM stage and cirrhosis), but not with patient survival [130].
miR-183	Oncogene	Hepatocellular carcinoma (serum)	--	Associated with prognosis (TNM stage and postoperative survival) [180].
miR-183	Tumor suppressor	Lung cancer (serum) *	--	Associated with prognosis (metastasis) [137].
miR-183	Tumor suppressor	Osteosarcoma (tissue)	EZR	Associated with aggressiveness and poor prognosis (tumor grade, response to chemotherapy, metastasis and recurrence) [135].
miR-183	-	Prostate cancer (cancer cell)	KLK3/PSA	miR-183 binds to the 3′ UTR of PSA and increases its protein and mRNA levels [136].
miR-96	Oncogene	Prostate cancer (tissue)	--	Associated with prognosis (tumor stage, recurrence and survival) [138].
miR-96	Oncogene	Prostate cancer (tissue)	--	Not correlates with prognosis (biochemical recurrence and clinicopathological parameters) [139].
miR-96	Oncogene	Hepatocellular carcinoma (tissue)	LRP6, FOXO1A, and MAP2K1 (Not biologically validated)	Associated with prognosis (recurrence) when combined with other microRNAs [140].
miR-96	Oncogene	Colorectal cancer (tissue) **	--	Associated with prognosis (overall survival)[32]
miR-96	Tumor suppressor	Colorectal cancer (plasma) **	KRAS	Associated with prognosis (distant metastasis and survival) [181].
miR-96		Acute myeloid leukemia (mononuclear cells)	--	Associated with prognosis (relapse-free survival and overall survival) [141].
miR-182	Oncogene	Nasopharyngeal carcinoma (tissue)	--	Associated with prognosis (overall survival, disease-free survival, and distant metastasis) [144].
miR-182	Oncogene	Pancreatic cancer (plasma)	--	Associated with prognosis (Clinical stages, lymph node metastasis and survival) [143].
miR-182	Oncogene	Breast cancer (tissue)	--	Associated with prognosis (lymph node metastases and grade III occurrence) [142].
miR-182	Oncogene	Colon cancer (tissue)	FBXW7	Associated with prognosis (Survival) [145].
miR-182	Oncogene	Colorectal cancer (tissue)	--	Associated with prognosis (T-stage, lymph node metastasis, distant metastasis, Dukes' stage, and survival) [146].
miR-182	Oncogene	Colorectal cancer (tissue)	--	Associated with prognosis (TNM stage, lymph node metastasis, and survival) [147, 148].
miR-182	Oncogene	Prostate cancer (tissue)	--	Associated with prognosis (T stages, Gleason score, TMPRSS2-ERG status, and patient survival) [149].
miR-182	Oncogene	Bladder cancer (tissue)	--	Associated with prognosis (aggressiveness and survival) [150].
miR-182	Oncogene	Glioma (tissue)	--	Associated with prognosis (overall survival) [151].
miR-182	Tumor suppressor	Lung cancer (tissue)	--	Associated with prognosis (disease-specific survival) [152].

* Contradictory findings were marked as the same number of *.

Abbreviations: EZR: Ezrin; FBXW7: F-box and WD repeat domain containing 7, E3 ubiquitin protein ligase; FOXO1A: Forkhead box O1a; KLK3/PSA: Kallikrein-related peptidase 3; KRAS: Kirsten rat sarcoma viral oncogene homolog; LRP6: Low density lipoprotein receptor-related protein 6; MAP2K1: Mitogen-activated protein kinase kinase 1

The high expression of miR-96 in prostate cancer is well documented [23, 41, 63, 66, 67, 138]. Larne *et al.* recently reported a miRNA index quote (miQ) in prostate cancer, which uses four miRNAs (miR-96, 183, 145, and 221) for more accurate diagnosis (area under the curve, AUC = 0.931) and prognosis (AUC = 0.895 for predicting aggressiveness and AUC = 0.827 for metastasis). miQ was verified in an independent Dutch cohort and three external cohorts, and significantly outperformed the prostate-specific antigen [131]. Schaefer *et al*. demonstrated that highly expressed miR-96 can predict cancer recurrence after radical prostatectomy [138]. Additionally, Haflidadottir *et al*. found miR-96 expression correlated with WHO grade, and the overall survival time in prostate cancer [67]. In contrast, a recent investigation found no significant correlation between the expression of miR-96 and clinicopathological parameters [139]. Thus, suggesting that more studies are required to understand the prognostic relevance of miR-96. In addition, miR-96 was reported as a potential biomarker for the predicting recurrence after surgical resection of hepatocellular cancer [140], and as prognostic indicator in lung cancer, colorectal cancer and acute myeloid leukemia [32, 48, 141] (Table 3).

Corresponding to the biological functions of miR-182 in various tumors, the up-regulation of miR-182 was associated with poor prognoses in hepatocellular carcinoma [13], breast cancer [142], pancreatic cancer [143], oropharyngeal carcinoma [144], colorectal adenocarcinoma [145–148], prostate cancer [149], bladder cancer [150], and glioblastoma [151] (Table 3). In contrast, the up-regulation of miR-182 was found to correlate with good prognosis in lung cancer [152]. We presume that this might be associated with the miR-183 target genes, such as RGS17, RASA1, CTTN, and FOXO3, which have been shown to inhibit cell proliferation, tumor invasion and metastasis in lung cancer cells [16, 57, 58, 153].

## CONCLUDING REMARKS

Recent studies suggest an important role of miR-183-96-182 cluster in tumorigenesis, cancer progression, tumor invasion and metastasis. Although most of the reports showed that miR-183-96-182 cluster is an oncogene cluster, it also functions as a TSG by inhibiting cell proliferation and metastasis in certain cancer cells. We hypothesize that the different results observed in expression and function of the miR-183-96-182 cluster may result from different underlying tissue types, different expression abundance of miR-183-96-182 or their target genes, differences between cell lines (Table 1–2), differences between cell line and tumor tissue, tissue and blood (Table 3), and differences between detecting methods used. Recent studies have also indicated diagnostic and prognostic relevance of the members of miR-183-96-182 cluster, either independently or collectively. These new data on the functions of miR-183-96-182 cluster in various tumors suggest that further studies will be needed to clarify its functions in the various stages and histological subtypes in different types of tumors, which will significantly improve the accuracy of the prediction for tumor diagnosis or prognosis. As regards the conflicting results in certain tumors, we believe that miR-183-96-182 cluster might play different roles because of tumor heterogeneity, which will be important for the individual diagnosis and prognosis in anti-tumor treatment.

## SUPPLEMENTARY MATERIALS FIGURES AND TABLES



## Abbreviations

miRNA: microRNA
TSS: transcriptional start site
TSG: tumor suppressor gene
EMT: epithelial-mesenchymal transition.
