# Supplementary material for: Dysregulation and functional roles of miR-183-96-182 cluster in cancer cell proliferation, invasion and metastasis
Source: Oncotarget. 2016 Apr 12;7(27):42805–25. doi: 10.18632/oncotarget.8715 (PMC5173173; doi:10.18632/oncotarget.8715)

Dysregulation and functional roles of miR-183-96-182 cluster in cancer cell proliferation, invasion and metastasis

Supplementary Material

1-1. The outline of the manuscript.

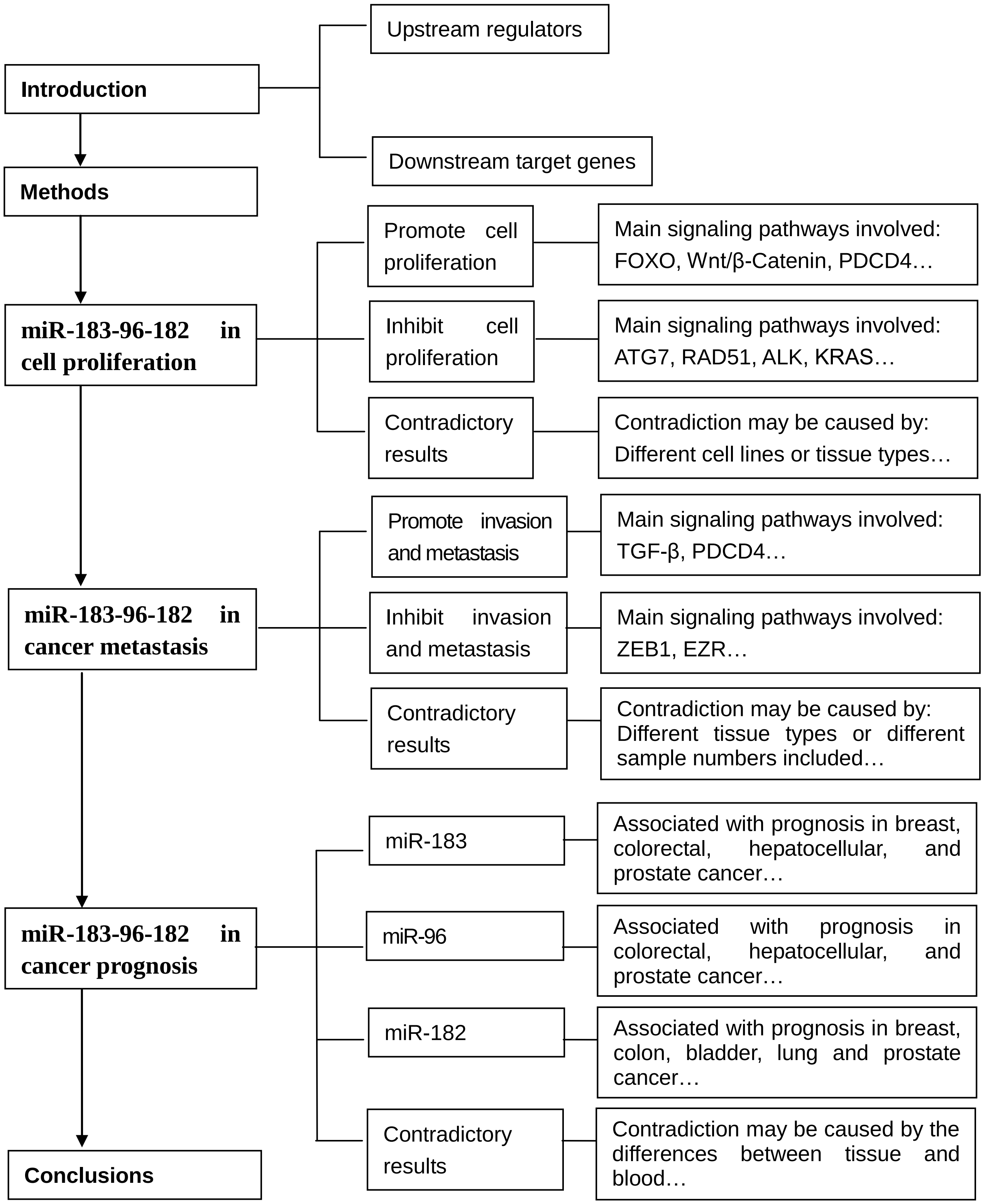

1-2. miR-183-96-182 cluster and WNT/beta-catenin form a positive feedback in promoting cancer cell proliferation.

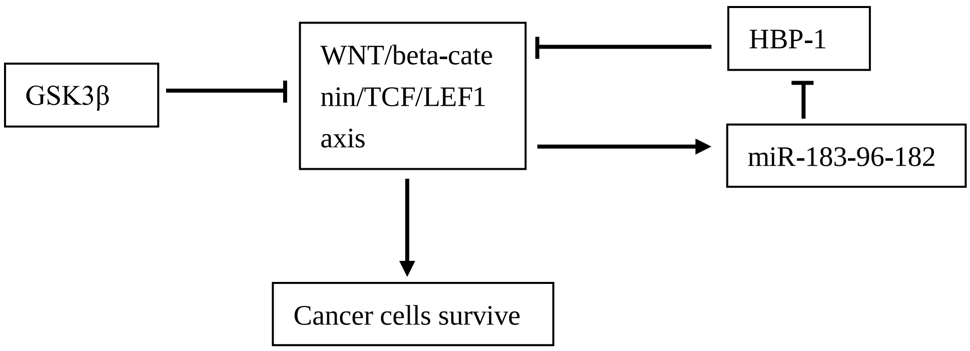

1-3. Some possible reasons for the reported contradictory results.

**Contradictory results**	**Possible reasons**
In the different tumor types	Differentially expressed miR-183-96-182 cluster<br>Differentially expressed target genes<br>Target genes with mutations<br>Other competing molecules exist<br>Involvement of different signaling pathways
In the same tumor type	Different cell lines<br>Heterogeneity of cancer tissue (e.g. different pathological grading)<br>Different experimental methods (e.g. CCK-8 or MTT)<br>Different experimental conditions (e.g. hypoxia or not)<br>Differences between in vitro and in vivo experiments<br>Differences between tissue and blood<br>Differences between cancer tissue and cell lines<br>Differences in study powers

1-4. miR-183-96-182 cluster and ZEB1 form a double negative feedback loop in inhibiting EMT.

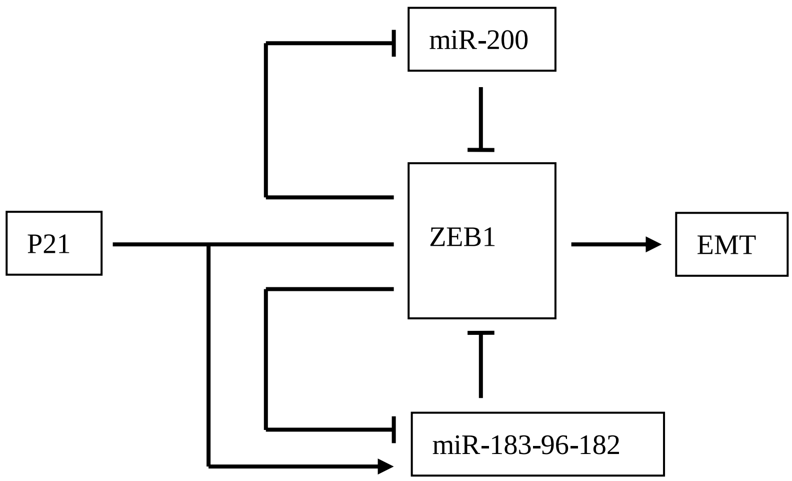

Supplement: Supplementary file 1 [file oncotarget-07-42805-s001.pdf]
